# Supplementary material for: Complexome profiling of the Chlamydomonas psb28 mutant reveals TEF5 as an early PSII assembly factor
Source: Plant Cell. 2025 Mar 17;37(6):koaf055. doi: 10.1093/plcell/koaf055 (PMC12164590; doi:10.1093/plcell/koaf055)
Supplement: koaf055_Supplementary_Data [file koaf055_supplementary_data.zip › psb28_interactive.zip!/plots/intensity/qualityPlots/peptides/DHC9_Cre02.g141606.t1.1.html]

### Cre02.g141606.t1.1

Trivial name: DHC9  
SeqCoverage [median]: 1.00  
Mass: 464.74 kDa  
  
 

| Sequence | MissedCleavage | Mass [Da] | wtIntensity | muIntensity | Score |
| --- | --- | --- | --- | --- | --- |
| AINDVNLPK | 0 | 982.54 | 8313.30 | 0.00 | 68.62 |
| PADITEVKGMKSPPAGVR | 2 | 1851.99 | 0.00 | 17488.26 | 53.40 |
| WLLDSESAIRRTLHK | 2 | 1824.00 | 1447.14 | 17296.04 | 32.27 |
|  |  |  | 9760.4 | 34784.3 |  |
